# Virchow's node together with an Irish node

**DOI:** 10.1002/ccr3.967

**Published:** 2017-04-26

**Authors:** Ezekiel Wong Toh Yoon, Kazuki Nishihara

**Affiliations:** ^1^ Department of Internal Medicine (Gastroenterology)Hiroshima Kyoritsu HospitalHiroshima CityJapan

**Keywords:** Gastric carcinoma, left axillary node, left supraclavicular adenopathy

## Abstract

Classical lymphadenopathies, such as Virchow's node (left supraclavicular lymph node metastasis) and Irish node (left axillary lymph node metastasis), are important findings that may indicate the presence of gastric cancer and other gastrointestinal malignancies.

A 95‐year‐old man presented with a 3‐week history of anorexia, nausea, and abdominal pain. Apart from tenderness in the epigastric region, physical examination also revealed enlarged, hard lumps in the left supraclavicular fossa and the left axillary region. Computed tomographic imaging confirmed these as a left supraclavicular lymph node metastasis (Virchow's node; Fig. 1A, yellow arrow) and a left axillary lymph node metastasis (Irish node; Fig. 1B, yellow arrow) respectively.

**Figure 1:**
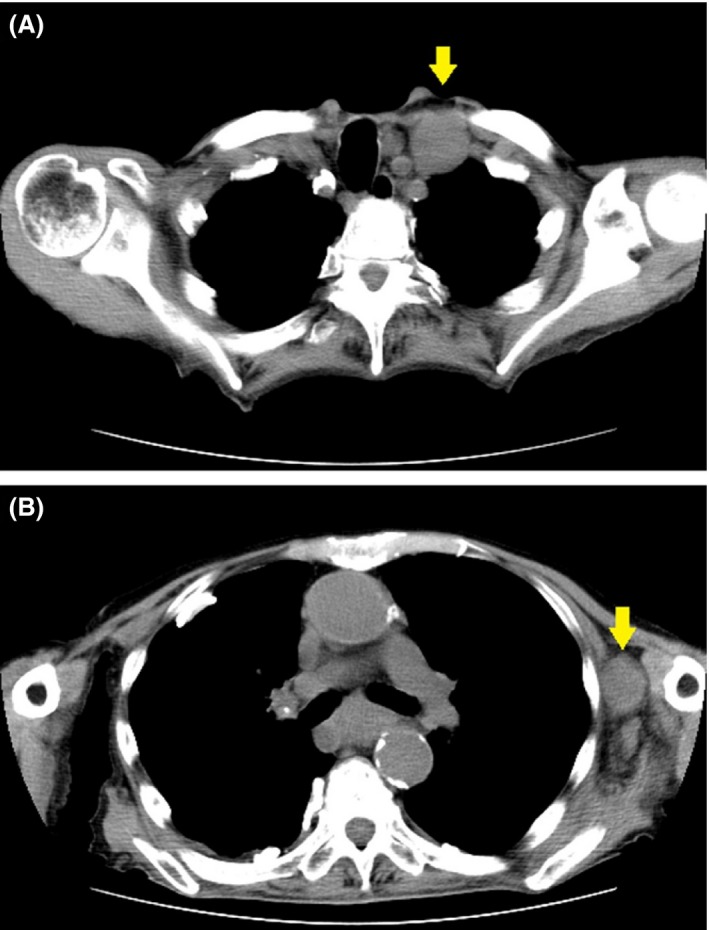
CT imaging revealed a left supraclavicular lymph node metastasis (A, yellow arrow) and a left axillary lymph node metastasis (B, yellow arrow).

## What was the cause of his symptoms?

**Figure 2:**
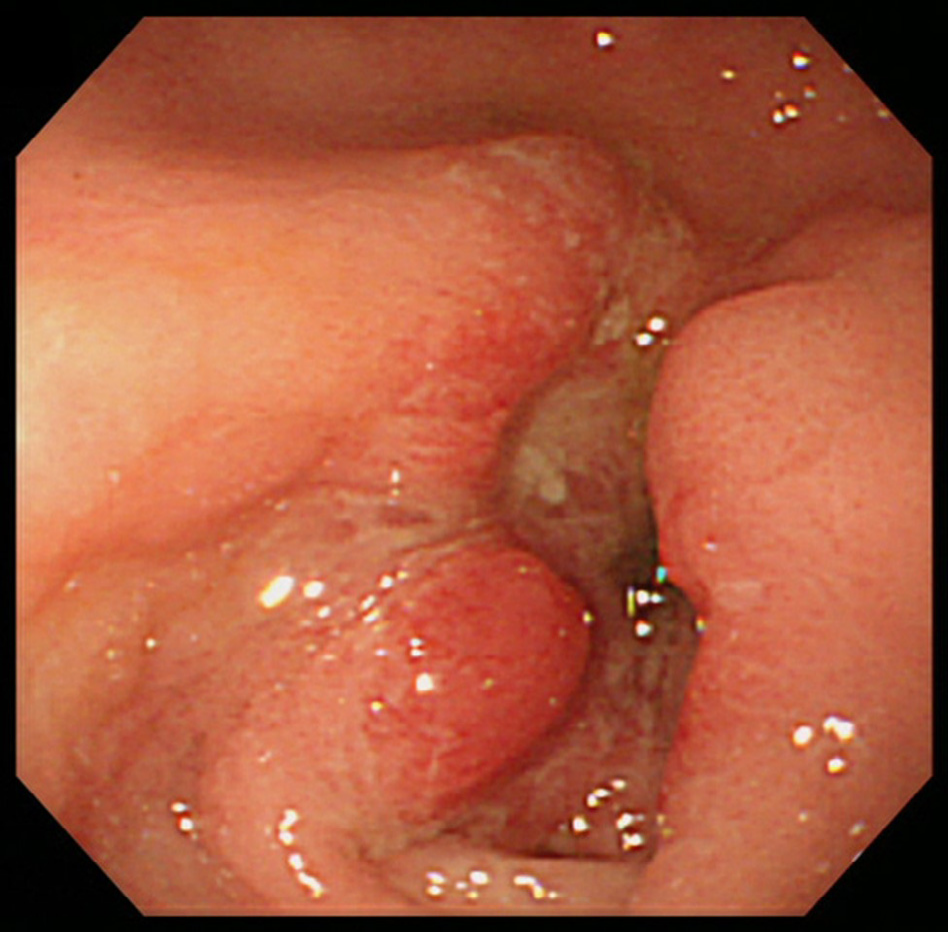
Endoscopy image showing ulceroproliferative growth in the antrum of the stomach.

Upper gastrointestinal endoscopy showed ulceroproliferative growth in the antrum of the stomach, and biopsy results were consistent with adenocarcinoma (Fig. 2). The patient underwent stenting to alleviate his symptoms and was discharged with full oral intake. Lymphadenopathies such as Virchow's node are important clues for gastrointestinal malignancies 1. The Irish node, although less common, can occur either together with the Virchow's node or as a rare solitary metastasis 2.

## Authorship

EWTY: prepared the manuscript. KN: had an advisory role in the management of the patient.

## Conflict of Interests

The authors have no competing interests to declare.
